# A Novel Accelerometry Method to Perioperatively Quantify Essential Tremor Based on Fahn–Tolosa–Marin Criteria

**DOI:** 10.3390/jcm12134235

**Published:** 2023-06-23

**Authors:** Annemarie Smid, Rik W. J. Pauwels, Jan Willem J. Elting, Cheryl S. J. Everlo, J. Marc C. van Dijk, D. L. Marinus Oterdoom, Teus van Laar, Katalin Tamasi, A. M. Madelein van der Stouwe, Gea Drost

**Affiliations:** 1 Department of Neurosurgery, University Medical Center Groningen, University of Groningen, Hanzeplein 1, 9713 GZ Groningen, The Netherlands; r.w.j.pauwels@umcg.nl (R.W.J.P.); j.m.c.van.dijk@umcg.nl (J.M.C.v.D.); d.l.m.oterdoom@umcg.nl (D.L.M.O.); k.tamasi@umcg.nl (K.T.); g.drost@umcg.nl (G.D.); 2 Department of Neurology, University Medical Center Groningen, University of Groningen, Hanzeplein 1, 9713 GZ Groningen, The Netherlands; j.w.j.elting@umcg.nl (J.W.J.E.); c.s.j.everlo@umcg.nl (C.S.J.E.); t.van.laar@umcg.nl (T.v.L.); a.m.m.van.der.stouwe@umcg.nl (A.M.M.v.d.S.); 3 Department of Epidemiology, University Medical Center Groningen, University of Groningen, Hanzeplein 1, 9713 GZ Groningen, The Netherlands

**Keywords:** accelerometry, essential tremor, quantification, algorithm, Fahn–Tolosa–Marin tremor rating scale

## Abstract

The disease status, progression, and treatment effect of essential tremor (ET) patients are currently assessed with clinical scores, such as the Fahn–Tolosa–Marin Clinical Rating Scale for Tremor (FTM). The use of objective and rater-independent monitoring of tremors may improve clinical care for patients with ET. Therefore, the focus of this study is to develop an objective accelerometry-based method to quantify ET, based on FTM criteria. Thirteen patients with ET and thirteen matched healthy participants underwent FTM tests to rate tremor severity, paired with tri-axial accelerometric measurements at the index fingers. Analogue FTM assessments were performed by four independent raters based on video recordings. Quantitative measures were derived from the accelerometric data, e.g., the area under the curve of power in the 4–8 Hz frequency band (AUCP) and maximal tremor amplitude. As such, accelerometric tremor scores were computed, using thresholds based on healthy measurements and FTM criteria. Agreement between accelerometric and clinical FTM scores was analyzed with Cohen’s kappa coefficient. It was assessed whether there was a relationship between mean FTM scores and the natural logarithm (ln) of the accelerometric outcome measures using linear regression. The agreement between accelerometric and FTM scores was substantial for resting and intention tremor tests (≥72.7%). However, the agreement between accelerometric postural tremor data and clinical FTM ratings (κ = 0.459) was low, although their logarithmic (ln) relationship was substantial (R^2^ ≥ 0.724). Accelerometric test–retest reliability was good to excellent (ICC ≥ 0.753). This pilot study shows that tremors can be quantified with accelerometry, using healthy thresholds and FTM criteria. The test–retest reliability of the accelerometric tremor scoring algorithm indicates that our low-cost accelerometry-based approach is a promising one. The proposed easy-to-use technology could diminish the rater dependency of FTM scores and enable physicians to monitor ET patients more objectively in clinical, intraoperative, and home settings.

## 1. Introduction

Essential tremor (ET) is a common movement disorder, with typically a stable tremor frequency between 4 Hz and 8 Hz [1,2,3]. In clinical practice, a qualitative rating of tremor is used to guide therapy. On one hand, patient-rated scales such as the Quality of Life in Essential Tremor Questionnaire (QUEST) and visual analog scale (VAS) are used to assess quality of life and patient satisfaction [4,5,6,7]. On the other hand, tremor rating scales such as the Fahn–Tolosa–Marin Clinical Rating Scale for Tremor (FTM) are also used by clinicians to assess disease severity, progression, and treatment effect [8,9]. During awake stereotactic neurosurgery aiming to alleviate tremor, the FTM scale is used to score tremor severity. Although the FTM is not a purely objective [10] scale, with assessments that may vary between raters, its results are directly applied in patient management [11,12]. As such, there is a clear need for measurements that allow precise and objective monitoring of ET, providing reliable outcomes [13,14].

Nowadays, hospitals are equipped with transducers to perform quantitative tremor measurements, but these can be complex, costly, and time-consuming. Previous studies have shown that transducer-based methods can complement clinical and intraoperative assessments [15,16,17,18]. Still, regular and rater-independent assessment of tremors often lacks in clinical practice [13].

A promising and inexpensive technique to objectively quantify tremors is tri-axial accelerometry (purchase costs USD 10~30). Outcome measures such as peak frequency and tremor amplitude can be calculated from accelerometric data, which could be used to translate clinical scales—such as the FTM—to a rater-independent method [18,19,20].

Previous research has mainly focused on determining objective measures that correlate well with clinical outcome measures, instead of attempting to literally translate a rating scale to a sensor-based method [12,13,15,21,22]. Therefore, using the standardized FTM criteria, our new approach is to develop an objective accelerometry-based scoring algorithm. Because most of the FTM criteria focus on the tremor amplitude in centimeters, the same amplitude thresholds are applied to our novel accelerometry-based scoring algorithm. The use of scoring thresholds based on accelerometric measurements in healthy volunteers will aid in differentiating physiological tremors from symptomatic measurements [18]. This way, we will develop a novel transducer-based method to contribute to the clinical assessment of tremors [16].

The focus of this study is to translate FTM (part A) into an objective accelerometry-based tremor rating algorithm. In this pilot study, the agreement between accelerometric and clinical FTM scores is evaluated. The proposed technology could diminish the rater dependency of FTM tremor measurements and enable physicians to more objectively monitor patients with ET.

## 2. Materials and Methods

This research project was performed in a tertiary referral center for movement disorders. The study was granted exemption from requiring ethics approval by the local medical ethics review committee, as the protocol was not deemed clinical research with human subjects as meant in the Dutch Act on Medical Research Involving Human Subjects. The study was conducted in compliance with the Helsinki Declaration for human research. 

Two wired tri-axial accelerometers (MMA8452Q tri-Axis, Freescale Semiconductor, Inc., Austin, TX, USA) and LabVIEW v. 2017 (National Instruments, Austin, TX, USA) were used for data acquisition [18]. MATLAB v. 2022b (MathWorks, Natick, MA, USA) and IBM SPSS statistics v. 26 (International Business Machines Corporation, New York, NY, USA) were used for data analysis and statistical analysis, as previously described [18].

### 2.1. Study Participants

Between September 2020 and December 2021, patients with ET visiting the neurosurgical outpatient clinic (as part of the standard workup for stereotactic surgery) were included and matched with healthy participants (HPs) for gender and age. All participants were adults and provided written informed consent for participation. ET patients had undergone a video-assisted surface EMG accelerometric tremor registration to confirm the diagnosis [2]. None of the HPs had a current diagnosis of ET or any neurological disease.

### 2.2. Clinical FTM Measurements

All tremor measurements were performed at the outpatient clinic. The participant was sitting in a chair with armrests during all measurements. Tremor severity was assessed using a total of three FTM tests: postural (wing-beating), intention (finger-to-nose), and at rest (arms on the armrest, hands free from the armrest). These tests were performed once for each hand.

Video recordings of the hands were made during the patient measurements to assess tremor severity according to the FTM scale by four experienced raters (A.S., R.W.J.P., C.S.J.E., and A.M.M.v.d.S.). The raters were blinded for the accelerometric data.

### 2.3. Accelerometry Measurements

**Figure 1:**
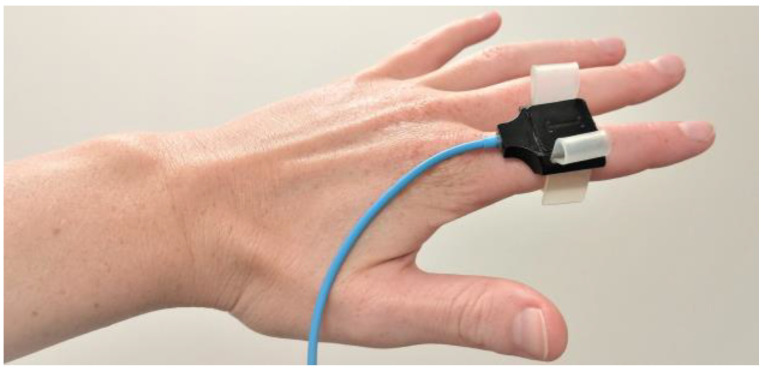
Accelerometer positioned on the left index finger.

Accelerometric data were recorded at the same time the clinical FTM tremor tests were performed for both hands. The sensors were placed at the proximal phalanx of both index fingers (Figure 1).

Accelerometry measurements in healthy participants were performed to estimate the naturally present level of noise in the 4–8 Hz frequency band and to compute thresholds of the accelerometry data for the scoring algorithm. Accelerometric data were recorded for ten seconds per test. Both hands performed the instructed posture (resting and postural) simultaneously, resulting in two (repeated) recordings per hand for each test. The intention tremor test was separately performed for each hand, so only data from the ipsilateral sensor were recorded during that test.

### 2.4. Accelerometric Outcome Measures

Data pre-processing was performed as described by Smid et al. [18]. Raw acceleration data were converted from g units to cm/s^2^. The root mean square of the resultant vector of the three axes was calculated in order to determine the acceleration norm [18]. To remove low-frequency noise, a non-causal second-order 0.5 Hz high pass Butterworth filter was applied. A non-causal second-order 20 Hz low pass Butterworth filter was used to suppress digital noise and higher-order harmonics [18,22]. To calculate the velocity (cm/s) per test, the approximate cumulative numerical integral of the filtered acceleration vector was computed over a time of ten seconds (2000 samples) via the trapezoidal method. The mean of the calculated velocity was subtracted from this vector to correct for the constant added during the integration step. Next, the corrected velocity was numerically integrated into the amplitude (cm). The amplitude vector was filtered using a non-causal second-order 1 Hz high-pass Butterworth filter to suppress the drift caused by integration. For the intention tremor tests, a cut-off frequency of 3 Hz was maintained to suppress the amplitude of low frequent arm movements [22].

To assess resting, postural, and intention tremor, two main outcome measures were calculated from the accelerometry data per respective test: (1) the area under the curve (AUC) of power within the 4–8 Hz tremor frequency band and (2) the maximal amplitude.

**Figure 2:**
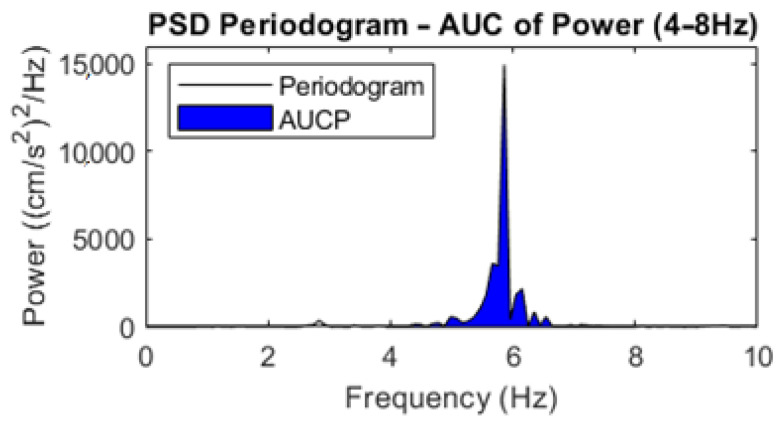
Example PSD periodogram, with the AUCP (4–8 Hz) given in blue.

From the periodogram power spectral density (PSD) estimate, the AUC of the power in the 4–8 Hz frequency band (AUCP: (cm/s^2^)^2^) was calculated via trapezoidal numerical integration (Figure 2) [18,22].

The maximal amplitude of each performed tremor test was calculated by multiplying the maximum value of the absolute amplitude vector by two, because a tremor is a sinusoid-like movement with equally negative and positive peaks.

### 2.5. Accelerometry-Based Scores

Objective tremor scores were computed from the accelerometry data, based on the standardized scoring criteria stated in the FTM scale (part A) [8]. The descriptive evaluations of the FTM were applied to calculate an accelerometry-based score via a custom scoring algorithm written in MATLAB. Similar to the FTM, the output of the scoring algorithm was a tremor score ranging from 0 to 4.

First, AUCP thresholds were calculated to differentiate healthy or asymptomatic measurements (FTM score of 0) from symptomatic measurements (FTM score ≥ 1). As the only criteria given for an FTM score of 0 is “none”, the AUCP of each test was calculated from the accelerometric HP data to determine the naturally present level of noise between 4 and 8 Hz. These natural levels were used to calculate thresholds that distinguish measurements of an FTM score of 0 from those of an FTM score ≥ 1. The Kolmogorov–Smirnov test of normality (one-sample K–S test) was used to determine whether the data were normally distributed. The thresholds for AUCP of the three different tremor conditions were defined as more than two standard deviations above the mean of the HP tremor tests.

Next, amplitude thresholds were calculated to distinguish between slight (FTM score of 1) and more severe (FTM score ≥ 2) tremors. The maximal amplitude of each test was computed from the accelerometric HP data. The thresholds were defined as more than two standard deviations above the mean amplitude of the HP tremor tests. These amplitude thresholds were separately calculated for the resting, postural, and intention tremor tests.

**Figure 3:**
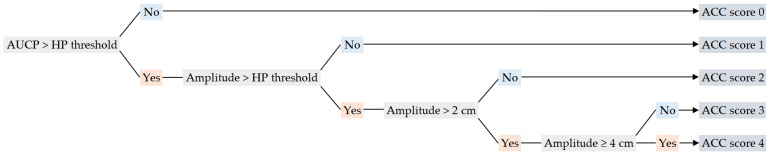
Flowchart of the accelerometry-based scoring algorithm, based on HP thresholds calculated from healthy participants (HPs) and standardized FTM scoring criteria. Following the flowchart, the “No” pathways are given in blue and the “Yes” pathways are given in orange.

If the AUCP of the test was below the determined threshold, an accelerometric (ACC) score of 0 was given (Figure 3). Otherwise, a score of 1 or higher was granted, based on the maximal amplitude measured during that test. If the maximal amplitude of the test was below the computed amplitude threshold, a score of 1 was granted. For the higher scores, the same amplitude limits described in the FTM scale were applied to calculate accelerometric scores (Figure 3).

### 2.6. Statistical Analysis

The ICC (two-way random effects model, mean of *k* raters, absolute agreement) of the four different raters was calculated to determine the inter-rater variability of the clinical FTM assessment [23]. The two-way random effects model with the mean of *k* raters approach was chosen here, as the inter-rater variability results are to be generalized to any raters who possess the same characteristics as the selected raters, and there was more than one rater.

Linear regression was used to analyze the relationship between the video-rated FTM scores and the two accelerometry outcome measures. For each test, the FTM scores provided by the four raters were averaged. The averaged FTM scores were used as the predictor and the outcome was the accelerometric outcome measure. Elble et al. described how the Weber–Fechner law of psychophysics postulates a logarithmic relationship between tremor ratings and tremor amplitude [24]. Therefore, it was assessed whether there was a linear relationship between mean FTM scores and the natural logarithm (ln) of the accelerometric outcome measures, as was also found in previous studies [15,17,18,24]. To this end, contrast coding of the mean FTM ratings was defined to be orthogonal polynomial, of which the first contrast coefficient is linear in trend [18].

Concordance and Cohen’s weighted kappa coefficient (κ) were calculated to measure the agreement between the mean FTM score and the accelerometric score of each test [25,26]. The mean absolute error (MAE) and the root mean square error (RMSE) were calculated to indicate how much the accelerometric scores differed from the mean FTM scores.

Lastly, the ICC (two-way mixed effects model, single measurement, absolute agreement) of AUCP, maximal amplitude, and accelerometric scores were calculated for the repeated resting and postural tremor tests in order to determine the test–retest reliability of the accelerometric approach [23]. The two-way mixed effects model with a single measurement approach was chosen here, as the selected measurements are the only measurements of interest, and the protocol was based on a single measurement and not on the mean of multiple measurements [23].

## 3. Results

Thirteen patients with ET (9 men, 4 women; age (mean ± SD): 73 ± 5 years) and 13 healthy participants (9 men, 4 women; age (mean ± SD): 73 ± 8 years) were included in this study. In the patient population, 44 resting, 44 postural, and 44 intention tremor tests were performed. A total of 78 measurements were performed in the healthy group. Median tremor severity in the patient population was an FTM (part A) score of 10 (range: 2–19) out of 24.

### 3.1. Clinical FTM Measurements

The ICC index between the four FTM raters showed good to excellent inter-rater reliability for all tremor tests (ICC ≥ 0.893) [23]. The ICC for the resting tremor test was 0.935 (95% CI: 0.855, 0.970; *p* < 0.001). For the postural tremor test, an ICC of 0.953 (95% CI: 0.918, 0.975; *p* < 0.001) was found. The ICC for the intention tremor test was 0.893 (95% CI: 0.813, 0.944; *p* < 0.001). Lastly, the ICC for all tremor tests combined was 0.959 (95% CI: 0.941, 0.972; *p* < 0.001).

### 3.2. Accelerometric Outcome Measures

**Figure 4:**
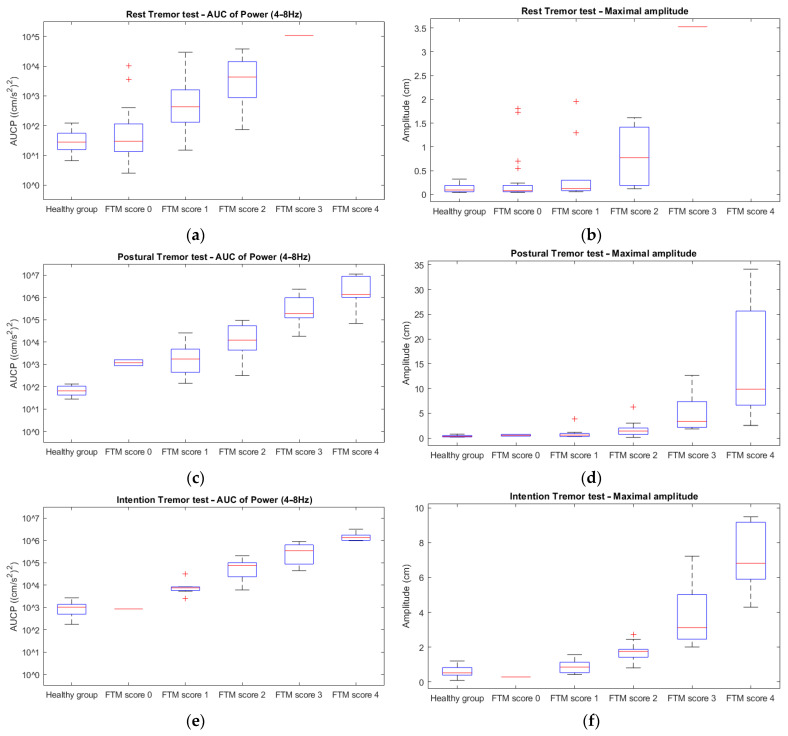
Boxplots of the AUCP between 4 Hz and 8 Hz of the resting (**a**), postural (**c**), and intention (**e**) tremor tests, and of the amplitude of the resting (**b**), postural (**d**), and intention (**f**) tremor tests in the healthy participants and the patient group (per FTM score).

The main outcome measures were the AUCP and maximal amplitude of the resting, postural, and intention tremor tests. In Figure 4, the quantitative outcome measures (AUCP and amplitude) of each type of tremor test are plotted against the averaged clinical FTM ratings. An increase in both AUCP and amplitude with increasing FTM scores can be observed for all tremor tests. These relationships tend to be logarithmic.

The main outcome measures were the AUCP and maximal amplitude of the resting, postural, and intention tremor tests. These accelerometric measures were natural log (ln) transformed and regressed on the averaged FTM scores. There was strong evidence against the null hypothesis that the ln-transformed outcome measures were not linearly related to the mean FTM ratings (*p* < 0.001) for all tremor tests (Table 1). In short, there was evidence for a logarithmic (ln) relationship between these variables.

AUCP tended to increase as a logarithmic function of FTM scores (R^2^ ≥ 0.714) for the postural and intention tests. This relationship was less strong for the resting tremor tests (R^2^ = 0.642).

The accelerometric amplitude also tended to increase as a logarithmic function of FTM scores (R^2^ ≥ 0.729) for the postural and intention tests. Again, this relationship was less strong for the resting tremor tests (R^2^ = 0.408).

### 3.3. Accelerometry-Based Scores

**Figure 5:**
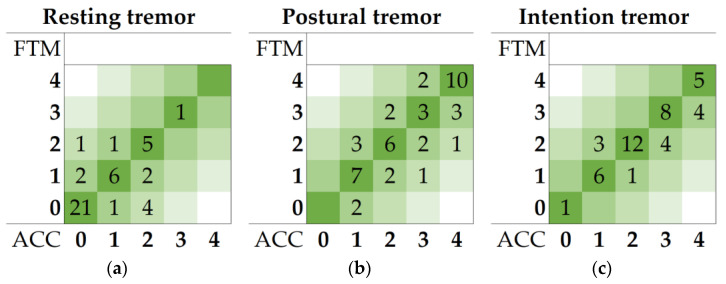
Contingency tables of the mean FTM scores and calculated accelerometry (ACC) scores of the resting (**a**), postural (**b**), and intention (**c**) tremor tests in the patient population.

No deviation from normality was detected by K–S tests for all HP data required for the threshold calculation. The AUCP threshold was used to differentiate FTM scores of 0 from FTM scores ≥ 1. An AUCP threshold of 123 (cm/s^2^)^2^ was calculated from the healthy population for the resting tremor test. This threshold was calculated to be 141 (cm/s^2^)^2^ and 2552 (cm/s^2^)^2^ for the postural and intention tremor test, respectively. The amplitude thresholds were used to differentiate FTM scores of 1 from FTM scores ≥ 2. The amplitude thresholds were calculated to be 0.32, 0.78, and 1.24 cm for the resting, postural, and intention tremor tests, respectively. The contingency tables of the averaged FTM scores and the ACC scores in the patient group for all types of tremor tests are given in heatmaps in Figure 5.

Concordance and Cohen’s κ coefficient of agreement between the averaged FTM scores and the ACC scores per type of tremor test, as well as the mean error (MAE and RMSE) are given in Table 2. Concordance between the FTM scores and the objective accelerometry scores was strong for resting and intention tremor tests (≥72.7%). Cohen’s κ results showed a moderate agreement for resting and postural tremors and a substantial agreement for intention tremor tests [25,26]. On average, the accelerometric scores differed less than half a point from the mean FTM ratings, as indicated by the MAE.

In Table 3, the test–retest reliability of the accelerometer-based method is given for the AUCP and amplitude outcome measures, and the calculated accelerometry scores of the repeated resting and postural tremor test. Reliability was excellent for the AUCP and amplitude of the postural tremor test, and the remaining ICCs showed good test–retest reliability [23].

## 4. Discussion

This pilot study focused on translating FTM tremor evaluations to objective accelerometry measurements. A scoring algorithm written in MATLAB to quantify tremors using tri-axial accelerometry measurements based on FTM criteria and HP thresholds was proposed. This study showed that tri-axial accelerometry is a promising technology for translating FTM tests. The association between tremor amplitude and FTM score was consistent with the logarithmic (ln) relationship shown in previous research [15,17,24].

The main contribution of this study is translating FTM measurements to an objective method, allowing clinicians to more uniformly rate patients. Although the inter-rater reliabilities found in this study were high, the validity of the FTM depends on the experience of the rater, underlining the need for an objective method [9,11,12,13,18].

Patients from the stereotactic surgery trajectory were selected in order to ensure the inclusion of high FTM tremor severity scores. Four experienced FTM raters were employed in order to provide reliable input for the comparison between clinical FTM scores and ACC scores. The FTM inter-rater variabilities found in this research were similar to the reliabilities of the FTM (part A) reported in previous studies [9,27].

The complex data gathering in our objective approach is presented in a way that is easy to interpret by physicians, as the ACC scores are based on the FTM criteria used in clinical practice. This research contributes to creating quantitative outcome measures in ET research and enabling physicians and other personnel to more objectively monitor patients with ET. Considering the few materials needed to perform the proposed accelerometric measurements, this technique is also suitable for monitoring tremors during awake neurosurgical procedures (e.g., deep brain stimulation surgery, MRI-guided focused ultrasound, and radioactive Gamma knife thalamotomy) [18,28,29,30,31]. As a future perspective, sensor-based measurement methods such as the one described in this article may allow patients to perform smartphone-assisted measurements at home, increasing patient self-management.

The trends between the clinical FTM scores and the AUCP, and between the clinical FTM scores and the maximal amplitude of the three different tremor measurements were in line with the findings of other studies [15,17,18,24]. This relationship was strongest for postural and intention tremor tests. Unsurprisingly, it was less strong for the resting tremor tests, considering the low number of resting tremor measurements with an FTM score above 2. This is to be expected in patients with a diagnosis of ET, as it is a phenotype that by definition is dominated by action tremor. This made the linear regression analysis more susceptible to the effects of outliers.

The unweighted agreement between accelerometric scores and FTM ratings was strong for resting and intention tremor tests (≥72.7%) [25]. However, a low agreement (59.1%) between accelerometric postural tremor data and clinical FTM ratings was found, although the linear relationship between ln-transformed accelerometric postural tremor amplitude and clinical FTM ratings was substantial (R^2^ ≥ 0.724). These discrepancies could be explained by objective measurements capturing small changes in amplitude that are difficult to visually evaluate. Continuous data are by definition capable of finer-grained distinctions than the discrete ordinal scale employed by the FTM, which is an advantage in monitoring disease progression [13,28]. However, the discrepancies could also be explained by the visual rating capturing aspects of the tremor that could not be measured at the position of the placed accelerometer, e.g., an isolated tremor in the thumb.

The accelerometric test–retest reliability was good to excellent (ICC ≥ 0.753) [23]. In both the resting tremor and postural tremor test, the test–retest reliability was highest for the AUCP outcome measure (ICC ≥ 0.887). This is explained by the large scale of AUCP values, in contrast to the 5-point scale of accelerometric scores. The test–retest reliability was lowest for the accelerometric score outcome in both resting and postural tremors. This is explained by AUCP and/or amplitude values being close to the calculated threshold, which makes the scoring algorithm sensitive to providing different scores for similar performances.

There are a number of limitations to this study. First, this study is a pilot study (proof of concept) with a relatively small sample size. In future research, this approach should be tested in a larger population in order to properly validate the proposed accelerometry-based approach. Future studies should also include out-of-sample validations in unseen data to avoid overfitting in the approach. In addition, to determine the test–retest reliability, there was only data available on the resting and postural tremor tests. Therefore, the reliability of the accelerometric approach was not assessed for the intention tremor tests. Moreover, the test–retest reliability was calculated based on two consecutive tests. It would be desirable to use three or more repetitions, preferably separated in time. As the test–retest reliability in clinicians was not investigated, a direct comparison between the reliability of the accelerometric approach and the FTM assessment was not possible.

In this study, the FTM scale was used as the gold standard for assessing ET severity. As this method relies on measurement by eye, this could explain the imperfect agreement found between accelerometric scores and clinical FTM ratings. Previous studies showed that visually estimated distances often do not agree with the distances objectively measured [21,24,32,33]. On the other hand, it is argued that the within-subject random variability of tremors from moment to moment makes it hard to lower the test–retest reliability of clinical assessments using transducers [16].

Third, the proximal phalanx of the index finger was chosen to measure hand tremors. If clinical raters primarily observe tremors in the more distal region of the index finger, or in a different part of the hand altogether, this will cause discrepancies in the agreement between the FTM and the accelerometric approach. In addition, the amplitude of tremors can differ between the proximal and distal positions of the hand [1,2,34]. Therefore, it is important to carefully consider possible sensor positions in future research.

Lastly, when clinicians rate according to the FTM, it is considered whether the tremor is intermittent (mild to moderate tremor may be intermittent (FTM scores of 1 and 2, respectively)). Tremor intermittency was not incorporated in the accelerometric scoring algorithm. Together with possible differences in observed tremor amplitude, these aspects might complicate the comparison between sensor-based and clinical assessments. These issues have to be taken into account when further validating this technique, with the goal to monitor tremors more objectively, both in the clinic and in the home setting.

## 5. Conclusions

This pilot study showed that tremors can be accelerometrically quantified based on healthy thresholds and clinical FTM criteria. The agreement between clinical FTM scores and accelerometric scores was substantial. The logarithmic relationship between accelerometric data and FTM ratings was in line with previous findings. The reported test–retest reliability of the ACC tremor scoring algorithm indicates that our accelerometry-based approach is a promising one. The proposed technology could be developed further in order to diminish the rater dependency of FTM tremor measurements and enable physicians to more objectively monitor patients with ET, both in various clinical settings—such as intraoperative monitoring—and in the home environment.

## Figures and Tables

**Table 1:** Regression analysis results.

FTM Test	Outcome Measure	R	R^2^ *	Coefficient **	95% CI	*p*
Resting tremor	Ln (AUCP)	0.801	0.642	8.042	5.716, 10.368	<0.001
	Ln (amplitude)	0.639	0.408	2.887	1.541, 4.233	<0.001
Postural tremor	Ln (AUCP)	0.851	0.724	7.765	2.908, 9.622	<0.001
	Ln (amplitude)	0.857	0.735	2.784	1.779, 3.790	<0.001
Intention tremor	Ln (AUCP)	0.845	0.714	5.884	4.444, 7.325	<0.001
	Ln (amplitude)	0.854	0.729	2.463	1.882, 3.044	<0.001

* Coefficient of determination; ** contrast coefficient testing for linear trend.

**Table 2:** Agreement results.

FTM Test	Concordance	Cohen’s κ	95% CI	RMSE	MAE
Resting tremor	75.0%	0.581	0.470, 0.692	0.77	0.36
Postural tremor	59.1%	0.459	0.334, 0.584	0.74	0.45
Intention tremor	72.7%	0.632	0.516, 0.748	0.52	0.27

**Table 3:** Test–retest reliability of accelerometric approach (ICC: two-way mixed effects model, single measurement, absolute agreement).

FTM Test	Outcome Measure	ICC	95% CI	*p*
Resting tremor	AUCP	0.887	0.793, 0.938	<0.001
	Amplitude	0.772	0.583, 0.875	<0.001
	ACC score	0.753	0.512, 0.856	<0.001
Postural tremor	AUCP	0.954	0.915, 0.975	<0.001
	Amplitude	0.936	0.875, 0.966	<0.001
	ACC score	0.890	0.799, 0.940	<0.001

## Data Availability

The data may be available upon reasonable request.
